# Measurement and optimization of perioperative risk among patients undergoing surgery for esophageal cancer

**DOI:** 10.1093/dote/doad062

**Published:** 2023-10-28

**Authors:** Jessie A Elliott, Emer Guinan, John V Reynolds

**Affiliations:** Trinity St. James’s Cancer Institute, Trinity College Dublin and St. James’s Hospital, Dublin, Ireland; Trinity St. James’s Cancer Institute, Trinity College Dublin and St. James’s Hospital, Dublin, Ireland; Trinity St. James’s Cancer Institute, Trinity College Dublin and St. James’s Hospital, Dublin, Ireland

**Keywords:** anesthesia, cardiopulmonary exercise testing, esophageal cancer, esophagectomy, nutrition, prehabilitation, surgery

## Abstract

Esophagectomy is an exemplar of complex oncological surgery and is associated with a relatively high risk of major morbidity and mortality. In the modern era, where specific complications are targeted in prevention and treatment pathways, and where the principles of enhanced recovery after surgery are espoused, optimum outcomes are targeted via a number of approaches. These include comprehensive clinical and physiological risk assessment, specialist perioperative care by a high-volume team, and multimodal inputs throughout the patient journey that aim to preserve or restore nutritional deficits, muscle mass and function.

## INTRODUCTION

Modern benchmark data from large series highlight that even in at a time of increased specialization, and centralization, and notwithstanding major advances in the approach to surgery and in perioperative care, surgery for esophageal cancer still presents a major challenge, with significant risks of major morbidity and mortality. Data from the Esodata.org database of over 6000 contemporaneous patients at 39 international centers, using strict definitions of complications developed by the Esophagectomy Complications Consensus Group (ECCG), report a 61% complication rate, and 30 and 90-day mortality in 2% and 4.5%, respectively. Complications have myriad consequences, including a longer inpatient length of stay, increased readmissions, a substantial increase in hospital costs, an increased risk of cancer recurrence, reduced long-term survival, and worse long-term health-related quality of life (HRQL).^1^^,^^2^ As such, the assessment and optimization of perioperative risk is critical to reduce the incidence postoperative morbidity and improve long-term patient outcomes.

**Fig. 1:**
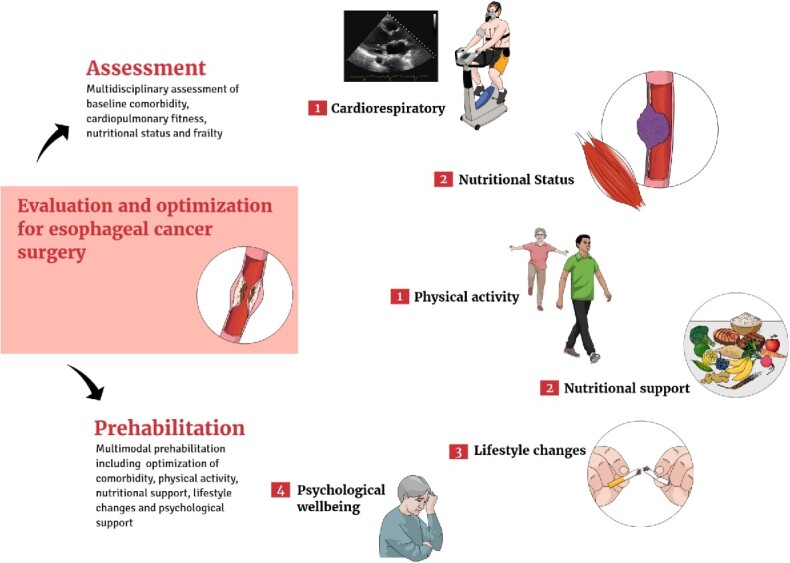
Measurement and optimization of perioperative risk among patients undergoing esophagectomy for esophageal cancer.

An assessment of perioperative risk encompasses a comprehensive review of patient comorbidity, nutritional and psychological status, and cardiopulmonary fitness. The most common tools utilized include cardiopulmonary fitness testing, and assessment of nutritional status and sarcopenia. The traditional approach has been to identify patients at high risk and to introduce measures to mitigate risk through optimization of co-morbid conditions, and by targeting nutritional deficiencies and physiological fitness through structured prehabilitation pathways. An evolving paradigm is that all esophageal cancer patients undergoing surgery irrespective of baseline health status are at risk of deteriorating health through treatment pathways that may include tri-modal therapy, or perioperative chemotherapy, accordingly interventional programs can be uniformly applied and not just selectively targeted. This article reviews the key elements of the modern approach (Fig. 1).

## PREOPERATIVE ASSESSMENT

### Medical assessment and comorbidities

Assessment of baseline medical comorbidities is important in assessing operative risk, to both identify prohibitive risks that should preclude surgery, and modifiable risks that can permit optimization within a relatively restricted time-frame. The American Society of Anesthesiologists’ score and European Co-operative Oncology Group performance status are global estimations of comorbidity and performance status, respectively, that have proven value in esophageal cancer cohorts.^3^^,^^4^

In esophageal cancer cohorts, smoking-related health problems are common, as are problems relating to obesity, particularly in males.^4–6^ In particular, visceral obesity, often associated with metabolic syndrome, including type 2 diabetes mellitus (T2DM), hypertension, hyperlipidemia, and non-alcoholic fatty liver disease is relevant to both the pathogenesis of esophageal adenocarcinoma and also the operative risk.^7^^,^^8^ Patients with metabolic syndrome may also exhibit micro-albuminuria, endothelial dysfunction and a pro-inflammatory and pro-thrombotic state, which may be associated with increased perioperative risk. Obesity and metabolic syndrome are reported to increase complications including surgical site infections, anastomotic leak, and cardiovascular events.^6^ Although direct evidence is lacking, it is intuitive that preoperative optimization of complications of obesity, such as hypertension, hyperlipidemia, T2DM and obstructive sleep apnea, may represent an important strategy to minimize the risk of perioperative complications in this cohort.

Smoking is an established risk factor for esophageal cancer, with increased prevalence of chronic pulmonary disease observed in this patient cohort as compared with the general population.^9^^,^^10^ Patients with underlying chronic pulmonary disease may require specialist pulmonology input and optimization of bronchodilator therapy to improve cardiopulmonary reserve and reduce the risk of postoperative pulmonary complications (PPCs) postoperatively. With respect to venous thromboembolism (VTE), surgery, chemotherapy and radiation therapy can induce a pro-coagulant response, and clinical VTE is reported in ~8% of esophageal cancer patients, 2.5% in the preoperative period, and this risk is significantly increased by neoadjuvant therapy (Odds ratio (OR) 3.84).^11^

Underlying psychiatric comorbidity and mood disorders may also require special attention and may require preoperative psychiatric consultation, additional psychological support and optimization of psychotropic medications. Particular consideration must be given to the perioperative administration and absorption of psychotropic medications among patients with background psychiatric comorbidity,^12^ and an *a priori* agreed perioperative medication management plan may significantly allay patient concerns regarding deterioration in mental health in the perioperative period.

A tailored approach to the preoperative evaluation and optimization of medical comorbidities may improve postoperative outcomes. Multidisciplinary care and inter-specialty collaboration are essential in the preoperative evaluation of patients.

### Cardiopulmonary fitness

#### Clinical assessment of cardiac function

Cardiovascular comorbidity is common among patients with esophageal cancer, and abnormalities in cardiac function may be exacerbated by preoperative malnutrition, chemotherapy and thoracic irradiation.^13^ Patients with cancer-associated weight loss have been shown to exhibit ‘cardiac cachexia’ with reductions in left ventricular wall thickness and increased myocardial fibrosis, while hypertrophic remodeling, or induction of restrictive cardiomyopathy has been observed with some chemotherapeutic agents, in particular anthracyclines.^14^ However, such changes may be nuanced and sub-clinical and undiagnosed at the time of surgery. In a clinical trial of non-small cell lung cancer, heart failure was observed on an echocardiogram in up to 14% of patients, however only 1.4% had a previously established diagnosis.^15^

Postoperative cardiovascular complications, including myocardial injury, can be predicted by the use of preoperative brain natriuretic peptide (BNP) levels. Several studies have demonstrated an association between preoperative BNP levels and the incidence of atrial fibrillation after thoracic surgery, with most studies including patients undergoing both major lung resection and esophagectomy.^16^ In one study, including only patients undergoing esophagectomy, a preoperative BNP level above the upper limit of normal was associated with a 4.7-fold increased risk of postoperative atrial fibrillation.^17^ Assessment of baseline BNP levels may represent a useful adjunct, and may complement a thorough preoperative evaluation including electrocardiogram and echocardiogram, which are increasingly standard for patients with esophageal cancer being considered for surgery, with further assessment tailored to pre-existing cardiac history, and findings of baseline assessment. For patients with identified abnormalities on baseline cardiac evaluation, early Cardiology consultation can be advocated, and in terms of mitigating risk, a multimodal approach, including pharmacological intervention and exercise training, is beneficial, and is further discussed in the following sections.^13^^,^^18^

#### Clinical assessment of respiratory function

A recent international collaborative series of 2704 patients from high-volume centers, with an approximate equal mix of open and minimally invasive approaches, demonstrated that PPCs were evident in 28% of patients, pneumonia in 15%, and respiratory failure in 7%.^19^ In other series, respiratory failure is reported in up to 15% of patients and is the most common cause of mortality.^19^^,^^20^ Prediction of risk and prevention of respiratory morbidity is therefore of considerable importance, and in this context baseline determination of lung physiology compliments clinical assessment. With respect to simple spirometry testing, a forced expiratory volume in the first second (FEV1) of ≥2.37 L was independently associated with a decreased risk of severe postoperative complications in a previous European multicenter study.^4^ Interpretation of spirometry values should take baseline patient factors such as age, sex, and height into account, and comparison to predicted values, which may be calculated according to the Global Lung Function Initiative (GLI) formulae, may be more valuable than application of distinct thresholds across a broad population.^21–25^

Importantly, there is increased evidence that neoadjuvant therapy, both multimodal therapy and pre or perioperative chemotherapy, can reduce FEV1 and forced vital capacity (FVC).^26–30^ However, more detailed pulmonary physiology studies have established that the most pronounced impact of these therapies is on pulmonary diffusion capacity, as measured by DLCO.^27^ The impact of radiation therapy combined with chemotherapy is more pronounced. Although radiation induced pneumonitis or fibrosis is rarely seen,^27^ initially sub-clinical changes in DLCO post-treatment have been shown to predict PPCs and long-term HR-QL. Smoking also independently increased DLCO loss, an effect also reported in lung cancer, further emphasizing the need for smoking cessation in patients commencing multimodal protocols.^31^ Current evidence indicates that assessment of pulmonary physiology, with standard spirometry and diffusion capacity, in addition to cross-sectional thoracic imaging, should be considered as standard at baseline, with repeat assessment recommended following completion of neoadjuvant therapy. Previous data from our center demonstrated that a threshold post-treatment DLCO of <65.0% predicts prolonged intubation for respiratory failure with 90% specificity among patients undergoing open esophagectomy.^27^ In an era where esophageal cancer surgery will increasingly be performed by minimally invasive approaches (MIE) or robotic assisted (RAMIE), both of which may reduce PPCs compared with open traditionally approaches, detailed prospective studies on pulmonary physiology and further reducing PPCs are warranted.

#### Cardiopulmonary exercise testing

Cardiopulmonary exercise testing (CPET) involves the measurement of respiratory gas exchange using breath-by-breath analysis to determine oxygen uptake (VO_2_), carbon dioxide output (VCO_2_) and minute ventilation to provide a non-invasive, dynamic assessment of the cardiovascular, pulmonary, hematopoietic, neuropsychologic and skeletal muscle systems at rest and under stress. CPET measures the maximum oxygen consumed during exercise (VO_2_max), which is characterized by a plateau in oxygen consumption despite the increase in workload during exercise.

Cardiopulmonary fitness is an established indicator of postoperative outcome and surgical candidacy in thoracic and abdominal surgery.^32^^,^^33^ In intra-abdominal surgery, low cardiopulmonary fitness, characterized by an anaerobic threshold (AT) <11 mL/kg/minute provides the most accurate indicator of postoperative morbidity and hospital length of stay.^32^ Comparably, peak oxygen consumption (VO_2_peak) <10 mL/min/kg is predictive of mortality and serious morbidity following major thoracic operations.^33^ Although intuitive that these approaches would apply to esophageal cancer patients, data at this time are inconsistent. A comprehensive systematic review involving 13 papers and 1735 participants, reported weak relationships between measures of fitness, specifically AT and VO_2_peak, and postoperative mortality, as well as conflicting evidence regarding the relationship with postoperative morbidity and no association with hospital length of stay.^34^ A separate meta-analysis reported moderate correlations between preoperative VO_2_peak and AT with postoperative cardiopulmonary complications, unplanned ICU admissions and 1-year survival, but no association with non-cardiopulmonary complications and no discriminative preoperative value to determine risk status.^35^ A pooled analysis of individual patient data (*n* = 621 patients) derived from these seven articles reiterated the limited value of preoperative CPET to discriminate between high and low risk patient scheduled for esophagectomy.^36^ Research is ongoing in this field and emerging literature does report a prognostic value of CPET derived variables and postoperative cardiovascular events,^37^ postoperative mobidity^38^ and 2-year survival,^39^ however further validation is needed.

Interestingly, the deleterious impact of neoadjuvant chemo(radio)therapy on physical fitness is proposed as an independent predictor of outcome. An analysis 100 open and MIE cases in the UK reported significant reductions in VO_2_peak during neoadjuvant chemo(radio)therapy in both males (mean difference −3.9 [95% confidence interval (CI) −4.9 to−2.9] ml/kg/min) and females (−2.1 [−3.6 to−0.6] ml/kg/min), and that this loss in fitness was associated with 1-year survival.^40^ In a similar retrospective analysis of 91 patients treated with CROSS chemoradiotherapy and open esophagectomy in the Netherlands, declines in exercise capacity during chemoradiotherapy were strongly associated with postoperative pneumonia (odds ratio [OR] 0.94, 95%CI: 0.89–0.99).^41^ While further studies are needed, this line of investigation may prove to be more sensitive and relevant to preoperative risk assessment for complex, attritional, multimodal treatment pathways.

#### Other measures of physical fitness

Structured walking tests measure functional exercise capacity and can be used to predict cardiopulmonary fitness, however the evidence that these tests can stratify or identify high-risk patients for esophagectomy is limited.^42^ Recent studies have proposed that walking distances below a threshold of 350 m on an externally paced incremental shuttle walk test^43^ or a threshold of 480 m on the self-paced 6-minute walk test (6MWT)^44^ are predictive of survival outcomes and that slightly lower 6MWT distances (454 m) are predictive of postoperative morbidity,^45^ nevertheless these cut-points require external validation.

Hand grip strength (HGS) provides a surrogate measure of overall muscle strength, correlating strongly with multiple important patient outcomes including hospital length of stay, functional limitations, quality of life and mortality.^46^ Grip strength, measured by calibrated hand-held dynamometry, is a core component in the clinical assessment of sarcopenia and consequently there is an emerging interest in HGS as a prognostic indicator of operative outcome, with recent studies reporting consistent associations with postoperative pneumonia. In a recent cohort of 188 patients treated with both open and MIE, low HGS was a significant independent predictor of postoperative pneumonia in people aged ≥70 years only (OR 4.89, 95% CI 1.53–15.67) and proposed a HGS cut point 30.5 kg as predictive of postoperative complications in people aged ≥70 years.^47^ A similar retrospective review of 161 patients undergoing MIE, identified low preoperative HGS as an independent predictor of postoperative pneumonia (OR 1.21; 95% CI 1.08–1.35) with no associations observed between CT-derived low skeletal muscle index and postoperative outcome.^48^ Furthermore, preoperative chemo(radio)therapy has a negative impact on skeletal endpoints including grip strength,^49^ with a detrimental impact on treatment tolerance,^50^ and a recent report from the Netherlands suggest that this loss in HGS is also strongly associated with postoperative pneumonia (OR 0.88, 95% CI 0.81–0.95).^41^ While this literature is in its relative infancy, there is considerable theoretical, clinical, and practical rationale for embedding a simple, inexpensive and non-invasive HGS assessment, which takes into account age and gender, into standard pathways to advance this field of investigation.

#### Frailty

Frailty has a well-researched association with HRQL, and postoperative morbidity and mortality.^51^ A myriad of tools exist, perhaps the most readily applicable tool for clinical implementation is the Rockwood Clinical Frailty scale. The Rockwood scale assesses frailty with a numeric value from 1 to 9, utilizing a subjective assessment from the health care professional. A higher score indicates greater levels of frailty and physical impairment.^52^ Other tools include the Physical Activity Scale for the Elderly (PASE) score, the Program of Research on Integration of Services for the Maintenance of Autonomy 7 (PRISMA7)^53^ and the Strength, Assistance, Rising from chair, Climbing stairs, Falls (SARC-F) questionnaire.^54^ The PASE measures physical activity over the previous 7 days using a Likert scale and was developed for the assessment of physical activity levels in older adults in the community. The transformation of answers was performed according to the New England Research Institutes scoring manual. Scores are represented as values between 0 and 400, with a higher score indicating a higher level of physical activity.^55^ Routine assessment of frailty can be advocated, particularly for older adults, who are being evaluated for consideration of surgery for esophageal cancer. In the authors’ experience, the Rockwood Clinical Frailty Scale is particularly useful in clinical practice, and now forms part of our standard preoperative evaluation for patients being considered for esophagectomy. Importantly for patients with esophageal cancer, frailty may be dynamic and may improve significantly with nutritional intervention, prehabilitation, optimization of medical comorbidity, or an additional period of recovery following neoadjuvant treatment. As such, reassessment following a period of optimization may be advocated for patients initially considered too frail to proceed to surgery, where an element of reversibility, particularly in relation to nutritional status, is anticipated.

### Nutritional assessment

The first Enhanced Recovery after Surgery Society guidelines for perioperative care in esophagectomy were published in 2019, containing 39 expert consensus-based recommendations, and highlight the importance of baseline nutritional assessment among patients with esophageal cancer.^56^ Malnutrition, which may manifest in sarcopenia, fatigue and reduced exercise tolerance, is associated with adverse outcomes both during neoadjuvant therapy, and following surgical resection.^57–59^ A recent meta-analysis has demonstrated that sarcopenia is associated with increased risk of PPCs, anastomotic leak, and reduced overall survival among patients undergoing esophagectomy for esophageal cancer.^60^ Furthermore, dietician-delivered intensive nutritional support may attenuate preoperative weight loss and reduce the incidence of severe postoperative complications.^59^ In the esophageal cancer care pathway, with multimodal or perioperative chemotherapy increasingly standard, there exists a window of opportunity for correction, and prevention, of malnutrition.

#### Nutritional screening tools

A number of tools exist. The Nutrition Risk Score (NRS) may be determined clinically by assessing body mass index, recent weight loss, reductions in food intake, age and the presence or absence of critical illness. Pre-screening of these criteria allows for identification of patients at risk of malnutrition, and enable risk stratification (Table 1). The NRS is recommended by the European Society for Clinical Nutrition and Metabolism (ESPEN) for use among hospital inpatients.^61^ A previous randomized controlled trial has demonstrated that the use of the NRS for risk stratification and tailored nutritional management in the inpatient setting is associated with reductions in in-hospital mortality.^62^ The main limitation of the NRS is that it is primarily suitable for use in the inpatient setting. The Malnutrition Universal Screening Tool (MUST) is endorsed by ESPEN for use among patients in ambulatory care and community settings, as well as hospital in-patients (Table 2). Calculation of the MUST score involves assessment of recent unintentional weight loss, and reductions in food intake lasting at least 5 days. Screening using the MUST can identify patients at high risk of malnutrition requiring nutritional support.

**Table 1:** Nutrition risk score

***Pre-screening***			
Is the BMI of the patient <20.5 kg/m^2^			Yes
Did the patient lose weight in the past 3 months?			Yes
Was the patient’s food intake reduced in the past week?			Yes
Is the patient critically ill?			Yes
If yes to one of those questions, proceed to screening.			
If no for all answers, the patient should be re-screened weekly.			
***Screening***			
**Nutritional status**	**score**	**Stress metabolism (severity of the disease)**	**score**
None	0	None	0
Mild Weight loss >5% in 3 months OR 50–75% of the normal food intake in the last week	1	Mild stress metabolism Patient is mobile Increased protein requirement can be covered with oral nutrition *Hip fracture, chronic disease especially with complications* e.g. *liver cirrhosis, COPD, diabetes, cancer, chronic hemodialysis*	1
Moderate	2	Moderate stress metabolism	2
Weight loss >5% in 2 months OR BMI 18.5–20.5 kg/m^2^ AND reduced general condition OR 25–50% of the normal food intake in the last week		Patient is bedridden due to illness Highly increased protein requirement, may be covered with ONS *Stroke, hematologic cancer, severe pneumonia, extended abdominal surgery*	
Severe Weight loss >5% in 1 month OR BMI <18.5 kg/m^2^ AND reduced general condition OR 0–25% of the normal food intake in the last week	3	Severe stress metabolism Patient is critically ill (intensive care unit) Very strongly increased protein requirement can only be achieved with (par)enteral nutrition *APACHE-II > 10, bone marrow transplantation, head traumas*	3
**Total (A)**		**Total (B)**	
**Age**			
<70 years: 0 pt			
≥70 years: 1 pt			
**TOTAL = (A) + (B) + Age**			
≥3 points: patient is at nutritional risk. Nutritional care plan should be set up			
<3 points: repeat screening weekly			

BMI, Body mass index.

**Table 2:** Malnutrition universal screening tool

**BMI (kg/m** ^**2**^**)**		**Unintentional weight loss in the past 3–6 months**		**Acute illness with reduced food intake (estimated) for ≥ 5 days**
≥20	0	≤5%	0	No = 0
18.5–20.0	1	5–10%	1	Yes = 2
≤18.5	2	≥10%	2	
**Overall Risk for Malnutrition**				
Total	Risk	Procedure	Implementation	
0	Low	Routine clinical care	Clinic: weekly	
			Nursing home: monthly	
			Outpatient: yearly in at-risk patient groups, e.g. age > 75 years	
1	Medium	Observe	Clinic, nursing home, and outpatient:	
			Document dietary intake for 3 days.	
			If adequate: little concern and repeat screening (hospital weekly, care home at least monthly, community at least every 2–3 months).	
			If inadequate: clinical concern. Follow local policy, set goals, improve and increase overall nutritional intake, monitor and review care plan regularly.	
≥2	High	Treat	Clinic, nursing home, and outpatient:	
			Refer to dietitian, Nutritional Support Team, or implement local policy. Set goals, improve and increase overall nutritional intake. Monitor and review care plan (hospital weekly, care home monthly, community monthly).	

The Subjective Global Assessment (SGA) represents an assessment of the overall nutritional status of a patient, carried out by a trained clinical nutritionist, and includes information on a medical history (weight loss, dietary intake, gastrointestinal and functional impairment) and physical examination (loss of subcutaneous fat, muscle wasting, ankle or sacral edema, and the presence of ascites). Using the SGA, patients may be classified as either well nourished (SGA A), moderately or suspected of being malnourished (SGA B), or severely malnourished (SGA C). A limitation of the SGA is that it does not capture subtle nutritional impairment and is hence less useful in the setting of dynamic changes in nutritional status, as is frequently the case among patients with esophageal cancer. Furthermore, the SGA does not mandate the assessment of objective measures such as serum biochemistry or anthropometric measures, which may limit its precision and reproducibility.^63^

Accurate assessment and documentation of the degree of dysphagia is also essential among patients with newly diagnosed esophageal cancer. We currently utilize the Mellow and Pinkas dysphagia score, which classifies the extent of dysphagia as follows: 0, Able to eat a normal diet; 1, Able to eat some solid food; 2, Able to eat semisolids only; 3, Able to swallow liquids only; and 4, Complete dysphagia.^64^ The strength of this tool is its simplicity and accuracy, although it does not capture the wider breath of dysphagia related symptoms such as aspiration, or the quality-of-life impact of dysphagia. The Mayo Dysphagia Questionnaire-30 was designed specifically to assess esophageal dysphagia, and includes a broader assessment of dysphagia-associated symptoms, with good validity and reproducibility.^65^ Several dysphagia-specific patient-reported outcome measure tools are also available, predominantly useful in clinical research.^66^ Standardized assessment and documentation of dysphagia severity facilitates clinical communication and enhances multidisciplinary decision-making.

#### Anthropometric assessments

Body mass index (BMI) is commonly measured, however caution should be observed with the use of BMI alone to screen for malnutrition among weight-losing populations, such as patients with newly diagnosed esophageal cancer, who may exhibit features of sarcopenia and micronutrient deficiency despite a normal or raised BMI.^67^^,^^68^ Other straightforward and low-cost non-invasive methods are the measurement of the circumference of a limb (e.g. mid-upper arm, skinfold thickness), and of waist and hip circumference. These measurements require trained staff and are moderately time consuming, but are useful particularly in resource-constrained settings.

#### Body composition analysis

Although weight and BMI are at the core of most screening tools, these measures are not reliable in the assessment of malnutrition, representing both fat and muscle mass.^69^ A number of techniques have been widely used for the assessment of body composition, including bioimpedance analysis (BIA), dual-energy X-ray absorptiometry (DXA), magnetic resonance imaging (MRI) and computed tomography (CT).

BIA is a method for rapid, simple and non-invasive assessment of body composition. BIA is based on the principle that the human body is regarded as an electrical conductor in an alternating current circuit and its alternating current resistance (impedance) is measured. A low alternating current (AC) is conducted into the body via electrodes and the reduction in voltage is measured via a second electrode pair in each case, allowing the components of body impedance to be determined. If the AC is applied at different frequencies, specific parameters can be individually assessed. In this way, for example, the proportion of extracellular water can be determined directly if low frequencies are utilized. At this frequency, AC penetration of cell walls is minimal, and as such cell walls and intracellular water exert minimal impact on impedance allowing estimation of extracellular water volume. Using the measured parameters in combination with the weight, height, age, sex, and activity level of a patient, features of body composition may be determined. BIA is inexpensive, convenient, accessible and safe and does not expose patients to radiation. It is not subject to operator bias or impacted by activity level.^70^ Overall, BIA approximates data achieved with more cumbersome gold standard methods such as DXA, CT, or MRI. However, BIA may lack sensitivity to detect small changes in body composition, may overestimate fat mass and underestimate lean body mass compared with DXA, and may be affected by hydration status and recent dietary intake.^71^^,^^72^ Furthermore, BIA is not suitable for patients with electronic implants such as pacemakers or other active prostheses or portable electronic devices. As such, BIA may be considered suitable for classification of a population in a research context, particularly where large differences in body composition are anticipated, however significant intra-individual variability in measurements limits the clinical utility of BIA.

In patients with cancer, CT scans utilized for cancer staging and to assess responses can also be used for body composition analysis. Several software packages exist, and utilize automated algorithms based on assessment of fat and muscle cross-sectional area at various levels on CT imaging. Using these methods, a number of measures, including lean body mass (LBM), skeletal muscle index (SMI), and intramuscular adipose tissue (IMAT), visceral (VAT) and subcutaneous fat areas (SFA), may be determined. Thresholds have been established in other oncology populations for the definition of sarcopenia based on the SMI, and among patients with esophageal cancer for the definition of visceral obesity.^73–75^ CT measures of body composition correlate closely with those obtained by DXA, the gold standard, but without the requirement for additional scans; associated cost and appointment burden for patients.^75^^,^^76^ Despite its accuracy, the associated radiation exposure renders CT unsuitable as a tool for dedicated assessment of body composition, or for short interval nutritional response assessment, which is an advantage of MRI, BIA, and DXA.

Other commonly utilized modalities include DXA and MRI. DXA provides assessment of both whole body and regional bone mineral, fat free mass and fat mass and is a broadly accepted tool for body composition assessment. DXA scanning is associated with a small amount of radiation exposure (~1–10% of a single chest radiograph).^77^^,^^78^ The benefits of DXA include accuracy and reproducibility, while the limitations include radiation exposure, upper limits for body weight and increased error with increasing trunk size. Limitations of MRI include high costs and scanning times, persons with certain metallic implants or foreign bodies *in situ* cannot be scanned, persons with claustrophobia often cannot tolerate scanning, and as well as limits to cross-sectional size based on the scanner’s field of view.^77–79^

## PREHABILITATION

Cancer prehabilitation aims to reduce post-treatment morbidity and improve pre-treatment health status to increase treatment options, typically surgical candidacy, through targeted interventions aimed at modifying behavioral and lifestyle risk factors.^80–82^ Approaches may involve exercise, nutritional optimization, smoking cessation, alcohol modification, or management of psychological issues, centered on the patient’s needs. Mitigating preoperative risks through prehabilitation in advance of high-risk oncological resections is an attractive concept, however, significant challenges exist for implementation into practice.

### Optimization of medical conditions

For patients with a history of pulmonary disease, or with abnormalities detected on baseline pulmonary function testing, preoperative input from a Respiratory specialist is advocated. Similarly, patients with background cardiac comorbidity or newly identified valvular abnormalities, arrythmia or inducible ischemia, should undergo preoperative Cardiology assessment. Perioperative cardiac arrythmia is common, occurring in between 10- 20% of patients undergoing esophagectomy.^83^ Current evidence supports continuing beta-blockers in the perioperative period in those who are on beta-blockers, and to prescribe beta-blockers for high-risk patients with coronary artery disease undergoing high-risk non-cardiac operations.^84^ Acute withdrawal of aspirin as secondary prevention in the perioperative phase may result in increased risk of coronary and cerebrovascular events among patients undergoing esophagectomy, and in such cases aspirin therapy should not be routinely interrupted perioperatively.^85^ Prophylactic amiodarone may reduce the incidence of postoperative atrial fibrillation but current evidence does not support a significant reduction in length of stay, overall morbidity or mortality in patients undergoing esophagectomy.^56^ Perioperative cardiac rhythm management strategies should be patient specific, aimed to reduce the modifiable risk factors and prompt recognition and treatment of associated or contributory complications.^56^ The American College of Cardiology/American Heart Association guidelines for perioperative cardiovascular evaluation in non-cardiac surgery provide thorough guidance regarding perioperative cardiac risk evaluation and management.^84^ These guidelines include evidence-based recommendations for the evaluation of perioperative cardiac risk, implementation of perioperative risk-reduction strategies, anesthetic management and postoperative monitoring, and provide a useful reference for the perioperative management of patients undergoing esophagectomy for cancer.

### Physical prehabilitation

Physical prehabilitation typically includes a range of interventions comprising aerobic, resistance and/or respiratory exercises. In intra-abdominal surgery, prehabilitation involving inspiratory muscle training (IMT), aerobic exercise and resistance training reduces the incidence of postoperative complications by 41% (OR 0.59 [95% CI 0.38–0.91]), with the strongest impact observed in relation to postoperative complications specifically.^86^ In esophageal cancer, two recent systematic reviews, one reviewing multimodal interventions involving a physical component^87^ and another examining physical prehabilitation as a unimodal intervention^88^ both highlight the issues with heterogeneity of interventions and outcomes, poor study quality and the dearth of powered, well-conducted randomized trials, however the literature continues to emerge.

Inspiratory muscle training (IMT) aims to strengthen the inspiratory muscles using a hand-held breathing device that adds resistance to inspiratory efforts^89^ Preoperative IMT increases inspiratory muscle function, which is hypothesized to attenuate inspiratory muscle impairment postoperatively, potentially expediting recovery of postoperative lung function and protecting against PPCs. IMT can be completed by the patient unsupervised in their home and gains in inspiratory muscle strength occur relatively quickly (2–3 weeks) and therefore it is a feasible concept for esophageal cancer patients. Randomized trials (RCTs) of preoperative IMT in esophageal resection consistently report increases in preoperative inspiratory strength,^89–91^ however the translation of these gains to postoperative clinical outcome is unclear. An RCT comparing high-intensity IMT (*n* = 23) to a lower intensity endurance IMT protocol (*n* = 22) in the Netherlands, reported significantly lower incidence of PPCs (20% vs. 58%) and shorter hospital length of stay (LOS, 14 vs. 18 days) in the higher intensity group.^90^ However, in a far larger, multicenter international RCT (the PREPARE trial) comparing high-intensity IMT (*n* = 120) to usual care (*n* = 121), Valkenet *et al.* reported no difference in incidence of postoperative pneumonia, PPCs or hospital LOS.^91^ Furthermore, secondary analysis of the PREPARE data at this Dublin center observed no difference in postoperative mobilization or postoperative physical functionning.^89^

Structured exercise training involving aerobic and/or resistance exercise to increase cardiopulmonary fitness or muscular strength/endurance is a major component of physical prehabilitation, both as a unimodal intervention and as part of multimodal prehabilitation programs. Broadly speaking, exercise training programs can be categorized as those completed in the weeks between neoadjuvant treatment and surgery, and programs which commence at the start of neoadjuvant treatment and continue to surgery.

**Fig. 2:**
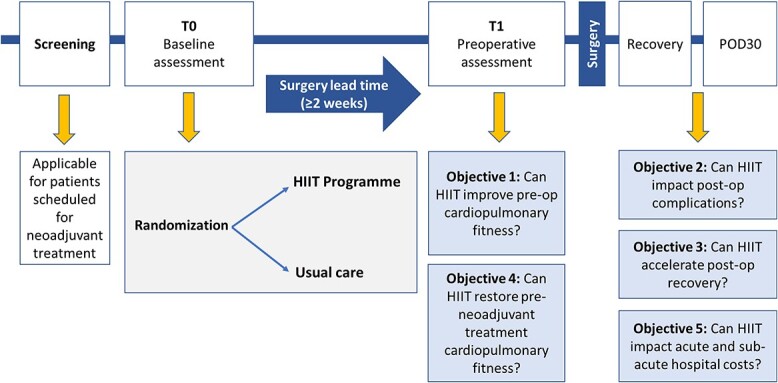
Design of the PRE-HIIT randomized controlled trial.

Evidence to support exercise training in patients undergoing surgery without neoadjuvant therapy or in the period between the end of neoadjuvant therapy and surgery is limited to small scale, single arm and retrospective reports supporting feasibility^92^ and suggesting some influence on postoperative functional capacity.^93^^,^^94^ One well-conducted, but small RCT reported that a preoperative home-based moderate intensity aerobic exercise program, performed over a median of 36 days, in combination with nutritional counseling, led to significant improvements in pre and postoperative 6MWT distance, but no impact on postoperative outcomes.^95^ Realistically, the time-frame to elicit change through aerobic or resistance exercise in the immediate preoperative period is limited in time-sensitive surgery and is a relative challenge for moderate intensity programs to elicit meaningful change in cardiopulmonary fitness.^96^ High intensity interval training (HIIT) may elicit greater change in physiological outcomes over a shorter period that may be required in high, risk complex oncological resections.^82^ An ongoing trial at this National Center, the PRE-HIIT trial (Fig. 2) will evaluate the feasibility and efficacy of a preoperative HIIT at increasing preoperative fitness in high complexity esophageal and thoracic surgery.^97^ In Australia, the PRIORITY TRIAL, will recruit 172 patients with a range of upper gastrointestinal cancer types and examine the effect of a community-based 12–24 session, multimodal physical prehabilitation program (exercise and respiratory exercises) on postoperative outcomes. Both of these adequately powered prospective RCTs will add greatly to the quality of literature in this field.

In patients undergoing multimodality therapy or perioperative chemotherapy, commencing prehabilitation at the start of neoadjuvant therapy expands the intervention period considerably. This is particularly relevant as these approaches have a negative impact on cardiopulmonary fitness and muscular strength,^27^^,^^42^^,^^98^ and prehabilitation during this period seeks to both prevent this decline and mitigate postoperative risk. At Imperial College London, the PREPARE program has consistently reported that multimodal prehabilitation, with moderate intensity aerobic exercise training a major component, abrogates the decline in cardiorespiratory fitness and positively influences postoperative outcomes, most particularly a 77% lower risk of postoperative pneumonia in comparison to patients who do not complete prehabilitation.^99–102^ This is mirrored in a UK RCT reporting that moderate intensity aerobic and resistance exercise training with individualized nutritional and psychological counseling attenuates the decline in cardiopulmonary fitness and loss of muscle mass, with resultant improved quality of life preoperatively.^103^ Treatment tolerance was also improved, with 75% in the treatment arm completing the full perioperative chemotherapy regimen, compared with 46% in the usual care arm. Similarly, in one report from the UK, higher rate of tumor regression were reported in patients completing moderate intensity aerobic exercise during treatment,^104^ another trial from Denmark suggested that those partaking in prehabilitation were more likely to progress to surgery.^105^ These aspects of prehabilitation require future review; however, the paradigm is consistent with the thesis that exercise during systemic therapy may improve treatment tolerance and reduce treatment dose adjustment, likely through preservation of lean body mass.^106^

Feasibility studies have reported excellent recruitment and retention rates to exercise programs during neoadjuvant treatment (up to 96%),^107^^,^^108^ however adherence to home-based interventions tends to be lower (68–76%).^103^^,^^105^^,^^109^ It is important that we identify and utilize strategies to optimize exercise participation during chemo(radio)therapy and use novel methods to understand adherence and exercise dose adjustment to inform clinical programs. Delivery of such exercise interventions may require intensive input from the multidisciplinary team, and the resource required may act as a barrier to implementation in clinical practice.

At present, a structured exercise intervention tailored to local resources, geographical factors, and clinical care models can be advocated among patients planned for esophagectomy. However, further research is needed to identify the cost–benefit of exercise interventions in this context, and to establish the core aspects of multimodal prehabilitation which deliver the greatest impact with respect to key outcomes including optimization of cardiopulmonary fitness, enhanced self-efficacy, and improvement in postoperative outcomes.

### Oral and enteral nutritional support

Malnutrition and micronutrient deficiencies are common among patients with esophageal cancer, and are associated with increased postoperative morbidity and mortality. Preoperative nutritional optimization represents an essential component of multimodal prehabilitation to improve clinical outcomes in patients with esophageal cancer. The optimal nutritional management strategy may be guided by the dysphagia score and the degree of nutritional compromise at presentation, as well as the patient’s social supports and home environment. For patients with minimal dysphagia, oral nutritional supplements may be recommended to supplement dietary intake, and education with a specialist clinical nutritionist regarding dietary modification strategies may be beneficial. For those with grade 2 or greater dysphagia, or clinically severe weight loss, enteral nutritional support can be generally recommended. Intensive nutritional support is associated with improved body weight and reduced postoperative complications among patients with esophageal cancer, with more marked effects observed among cohorts undergoing neoadjuvant therapy before surgery.^59^ The optimal route for enteral nutritional support has been widely debated.

The use of stents as a bridge to surgery has been shown to result in an early improvement in dysphagia scores, however meta-analysis has shown increased risk of margin positivity following esophageal stenting, and reduced overall survival.^110^ Furthermore, stent migration was reported in ~30% of patients undergoing neoadjuvant therapy.^111^ As such, stenting cannot be recommended as a routine strategy for nutritional optimization among patients being considered for curative intent resection for esophageal cancer.

Nasogastric feeding may be considered for patients who require home enteral nutritional support during neoadjuvant therapy. It is a low-risk intervention but is likely to be associated with a significant quality of life impact. Alternative options include percutaneous endoscopic or radiologically inserted gastrostomy, or laparoscopically inserted feeding jejunostomy. Few high-quality studies have compared outcomes according to the route of enteral nutritional support, and none have included comparison of patient-reported outcome measures. Meta-analysis has shown that home enteral nutrition is associated with improved BMI, lean body mass and skeletal muscle mass, physical and social function, and fatigue.^112^ Furthermore, home enteral nutrition was associated with reduced risk of subsequent postoperative pneumonia as compared with preoperative oral nutritional supplementation.^112^ Some authors have reported increased risk of small bowel obstruction among patients who have undergone feeding jejunostomy tube insertion, while a concern exists regarding the integrity of the gastric conduit for future esophageal reconstruction, as well as potential tumor seeding to the gastric wall, following gastrostomy placement. While optimization of protein-calorie malnutrition represents a critical target in modern prehabilitation protocols for patients with esophageal cancer, it is clear that considerable uncertainty remains regarding the optimal approach for enteral feeding support in this context.^111^

### Micronutrient supplementation

In addition to protein-calorie malnutrition, targeted treatment of micronutrient deficiency represents a key component of preoperative nutritional optimization for patients with esophageal cancer planned for surgical resection. Patients who have undergone treatment for esophageal cancer are at high risk of osteoporosis and fragility fracture in survivorship, with vitamin D deficiency a common finding on initial and follow-up assessments.^113^^,^^114^ Vitamin D is a fat-soluble vitamin that plays a crucial role in the regulation of calcium and phosphate metabolism. However, recent research has additionally highlighted the systemic effects of vitamin D deficiency, including increased risk of cardiovascular disease, autoimmune disorders, and infectious diseases.^115^^,^^116^ The active form of vitamin D has been found to modulate the immune system by promoting innate immunity and suppressing adaptive immunity. Additionally, vitamin D deficiency has been associated with impaired insulin secretion, leading to increased insulin resistance and the development of type 2 diabetes. In particular, the effect of vitamin D supplementation on postoperative atrial fibrillation, pneumonia and the systemic inflammatory response has been the focus of several recent clinical trials. Whereas smaller observational studies demonstrated an association between vitamin D deficiency and the occurrence of postoperative atrial fibrillation, larger cohort studies as well as RCTs assessing vitamin D supplementation could not confirm these results. A recent meta-analysis of patients undergoing coronary artery bypass grafting demonstrated a significant reduction in postoperative atrial fibrillation following supplementation of patients with baseline vitamin D insufficiency or deficiency (relative risk 0.60).^117^ The recent coronavirus-19 pandemic resulted in a revived interest in the potential effects of vitamin D supplementation in modulating the immune response to respiratory infection, with RCTs demonstrating possible benefit in terms of duration of symptoms.^118^^,^^119^ Preoperative vitamin D supplementation has also been shown to be associated with reduced postoperative C-reactive protein levels among patients undergoing surgery for colorectal cancer.^120^ Evidence regarding the impact of vitamin D supplementation on postoperative pulmonary infection and the systemic inflammatory response among patients undergoing esophagectomy is lacking, and further study in this area is warranted. Nonetheless, given the established benefits with respect to bone health, correction of vitamin D deficiency can be recommended as part of the routine nutritional management of patients with newly diagnosed esophageal cancer.

Treatment of anemia is clearly desirable among patients with newly diagnosed esophageal cancer, with vitamin B12 and folic acid supplementation generally successful in addressing these micronutrient deficiencies. The optimal approach for correction of iron deficiency among patients being assessed for esophageal cancer surgery remains unclear, with oral iron supplementation associated with prolonged recovery of hemoglobin levels, poor gastrointestinal tolerability, and the potential to mask ongoing gastrointestinal bleeding. The FIT trial assessed the use of oral versus intravenous iron for patients with iron deficiency anemia prior to elective colorectal cancer surgery.^121^ After commencing treatment a median of 14 days preoperatively, similar rates of normalization of hemoglobin were observed in both arms, however hemoglobin levels were significantly increased in the intravenous iron group at all postoperative timepoints. There were no significant differences in safety outcomes between groups. The PREVENTT randomized controlled trial investigated the effectiveness of preoperative intravenous iron therapy administered 10–42 days preoperatively to patients with anemia in reducing perioperative blood transfusions in patients undergoing elective major open abdominal surgery.^122^ The use of intravenous iron increased preoperative hemoglobin levels, but had no effect on risk of blood transfusion rates, postoperative complications, LOS or mortality. The intravenous iron group had higher hemoglobin levels at 8 weeks and 6 months postoperatively, and there was a significant reduction in re-admissions among patients treated with intravenous iron (rate ratio 0.54). Importantly only 20% of patients in PREVENTT had their anemia corrected before surgery, and similar to the FIT trial, hemoglobin levels continued to improve postoperatively, suggesting that earlier intervention may have resulted in better perioperative correction of anemia, of relevance in the context of neoadjuvant pathways in upper gastrointestinal cancer surgery. Further research is needed to evaluate the impact of correction of baseline anemia, including iron, B12 or folate deficiency, on perioperative outcomes among patients with upper gastrointestinal cancer.

### Immunonutrition

In recent years there has been an increasing interest in the role of functional foods and pharmaconutrition to optimize outcomes for patients with esophageal cancer. In particular, the role of immunonutrition using preparations enriched with immunomodulatory agents such as arginine, omega-3 unsaturated fatty acids and nucleotides, has been investigated. Trials assessing the impact of perioperative immunonutrition in esophagectomy have demonstrated conflicting results. While eicosapentaenoic acid (EPA)-enriched enteral nutrition preserved lean body mass and attenuated the stress response in comparison with standard nutrition in one RCT, two further RCTs demonstrated no significant difference in clinical outcomes with the addition of preoperative immunonutrition.^68^^,^^123^^,^^124^ There is currently insufficient evidence to support the use of immunonutrition as standard for patients undergoing esophagectomy outside of the clinical trial setting.

### Smoking cessation

Smoking is a significant risk factor for post-operative complications, associated with impaired wound healing, higher infection risk and significant cardiopulmonary complications.^125^ In patients undergoing esophagectomy, current smoking is an major independent risk factor for overall pulmonary morbidity (OR 1.47 [95% CI 1.08–2.01]) and postoperative pneumonia (OR 2.29 [95% CI 1.34–9.93]).^126^ Consequently, smoking status should be established preoperatively in all patients and smoking cessation encouraged, ideally through referral to smoking cessation support services.^82^ Longer duration of preoperative smoking cessation is associated with less severe postoperative complications following oesophagectomy^127^^,^^128^ and recent data suggests that smoking cessation >2 months is associated with enhanced short-term and long-term postoperative outcomes.^129^

### Alcohol modification

Alcohol consumption in excess of three alcoholic units per day or 21 alcoholic units per week increases postoperative complication rate by up to 50% and up 300% for people drinking >5 alcoholic units per day.^130^ Literature examining the effectiveness of perioperative alcohol cessation interventions are limited however for individuals engaged in risky alcohol consumption, intensive interventions involving pharmacological therapy may reduce postoperative complications in select cohorts.^131^ Preoperative assessment should establish weekly alcohol consumption with onward referral for specialist support if required.^82^

### Psychological support

There is increasing recognition that psychological factors associated with mood, attitude or personality may positively or negatively impact postoperative outcome. Favorable outcomes are associated with factors such as high self-efficacy, low pain expectations, external locus of control, optimism, religiousness and anger control, while unfavorable outcomes are associated with trait and state anxiety, depression, intramarital hospitality, state anger, and psychological distress.^132^ Dispositional optimism has been associated with improved outcomes following esophagectomy for cancer.^133^^,^^134^ There is limited evidence that preoperative interventions can attenuate or address psychological issues to positively impact surgical outcome; however, preoperative evaluation of psychological distress using validated outcome measures such as the Hospital Anxiety and Depression Scale (HADS) may be helpful. High-quality trials in this area are required.^82^

## SUMMARY

This review has examined the current strategies for preoperative assessment and optimization, including the use of risk assessment tools, nutritional support, and prehabilitation programs (Table 3). Comprehensive cardiorespiratory assessment is essential for patients under consideration for major resectional esophageal surgery, with pulmonary function testing, echocardiography and cardiopulmonary exercise testing central to this process. Assessment of patients with newly diagnosed esophageal cancer also mandates evaluation of nutritional status and dedicated tools for the assessment of malnutrition and frailty can facilitate a tailored and risk-based approach to preoperative optimization. Recent studies have highlighted the negative impact of sarcopenia and impaired physical function on perioperative outcomes for patients with esophageal cancer, and multimodal prehabilitation programs have a key role in attenuating muscle loss during neoadjuvant protocols, preserving strength and cardiorespiratory fitness, and attenuating treatment-related fatigue. Specialist nutritional management is associated with improved postoperative outcomes among patients with esophageal cancer undergoing esophagectomy, and emerging evidence suggests that micronutrient supplementation may be associated with improved outcomes following major abdominal or thoracic surgery, although data focusing on the esophageal cancer patient population are lacking.

**Table 3:** Assessment and optimization of perioperative risk among patients undergoing surgery for esophageal cancer

	**Assessment or intervention**	**Recommendation**
**Global evaluation**	European Co-operative Oncology Group performance status	A*
	American Society of Anesthesiologists’ score	A*
**Cardiopulmonary assessment**	Cross-sectional thoracic imaging	A*
	Electrocardiogram, echocardiogram	A
	Brain natriuretic peptide	C
	Standard spirometry	A
	Diffusion capacity for carbon monoxide	A
	Cardiopulmonary exercise testing	C
	Structured walking tests	C
	Hand grip strength	C
	Frailty scoring systems	C
**Nutritional assessment**	Malnutrition Universal Screening Tool	A*
	Nutrition Risk score	B
	Subjective Global Assessment	B
	Dysphagia scoring systems	A*
	Weight, height, body mass index	A*
	Other anthropometric measures	C
	Body composition analysis	C
**Preoperative optimization**	Inspiratory muscle training	D
	Structured exercise prehabilitation	C
	Tailored nutritional support	A*
	Immunonutrition	D
	Stenting as a bridge to surgery	D
	Vitamin D supplementation	C
	Strategies to correct anemia	C
	Smoking cessation	A
	Alcohol reduction interventions	C
	Psychological interventions	C
**Medication management**	Continuation of beta-blockers	A
	Continuation of aspirin as secondary prevention	A
	Prophylactic amiodarone	C

A, Evidence-based support for routine application; B, Evidence-based support for selective application; C, Parameters which have demonstrated potential relevance but require additional research; D, Parameters that have been assessed but current evidence cannot support their utilization at this time; A*, Recommended as best practice without established evidence base.

It is a dynamic time in clinical and scientific research on this topic, with a solid and rapidly evolving evidence-base established through trials and international collaborations, building on a compelling scientific hypothesis. Although interventional trials of physiotherapy and conditioning have focused primarily on aerobic health, and cardiorespiratory risk, there is an emerging focus on preservation of muscle mass which may involve the systematic assessment of resistance training in these populations of patients. Although research is in its relative infancy, what is clear is that already in 2023 sufficient evidence exists to support a multidisciplinary approach to address modifiable risk in all patients with esophageal cancer who require surgery as a component of their curative intent management.

## Notes

Category: Frontiers in Esophageal Diseases Review.

Reprints: Jessie A. Elliott, Trinity St. James’s Cancer Institute, Dublin 8, Ireland. Email: jelliott@tcd.ie
